# Title VI: Modernizing Allotments for Vulnerable Elder Rights Protection Activities and Other Programs

**DOI:** 10.1093/geroni/igaa057.2521

**Published:** 2020-12-16

**Authors:** Taneika Duhaney

**Affiliations:** Health and Aging Policy Fellowship, Springfield, Virginia, United States

## Abstract

Of the many changes to the OAA, the Modernizing Allotments for Vulnerable Elder Rights Protection Activities and Other Programs section included notable changes. This bill specifies that Title VII programs will receive a 7% increase in 2020 and a 6% increase in the following four fiscal years. It extends the Supporting Grandparents Raising Grandchildren Act for an additional year. The reauthorization ensures that ombudsman representatives can be reimbursed for costs incurred through their services. The Act requires that the Government Accountability Office study federal programs for home modification assistance for older adults and individuals with disabilities. The Act directs the Administrator of the Administration for Community Living to continue the 2017 requirement of disseminating and soliciting feedback on the Principles for Person-directed Services and Supports during Serious Illness. This reauthorization updates home and community-based best practices; and elder justice activities, including community outreach and education to bolster community partnerships.

